# Supplementary material for: Pregnancy Rates of Holstein Friesian Cows with Cavitary or Compact Corpus Luteum
Source: Vet Sci. 2024 May 30;11(6):246. doi: 10.3390/vetsci11060246 (PMC11209027; doi:10.3390/vetsci11060246)
Supplement: Supplementary file 1 [file vetsci-11-00246-s001.zip › vetsci-2965288-supplementary.pdf]

Supplementary File S1

Transrectal ultrasound examination in cows in the present retrospective study

Animals have been selected for pregnancy diagnosis on a weekly schedule generating a list with a 4-digit cow identification number (ear tag) downloaded from the farm management software each week. During the morning milking (06:00-08:00 CET), cows have been selected and marked with a red coloured animal chalk on the middle of the head and placed into a separate pen designed for insemination and pregnancy diagnosis on the farm.

An experienced operator with decades of ultrasound experience identified the cow and then emptied the rectal lumen manually. With a portable ultrasound equipment (General Electric Logiq V2) placed on its transfer cart, and endorectal probe was inserted into the rectum of the cow directed with the operator's hand and arm and the entire internal genital tract has been scanned as follows: first, the vagina, the cervix and the uterine body, then the left uterine horn with the left ovary with its structures and then the right uterine horn with the right ovary and its structures.

If foetal fluid has been found in the uterine lumen, the body of the embryo has been checked for a detectable heart pulsation and heartbeat. The entire uterine lumen has been scanned 3 times in a row to be certain in the findings. If neither embryo(s) nor remnants of pregnancy have been detected upon the examination, the entire uterus has been scanned 2 more times.

Ovaries on both sides have been scanned and the structures have been described for number and approximate size. Follicles, corpora lutea or ovarian cysts all have been noted as described by others (Pierson et al., 1988; Vanholder et al., 2006).When finding a corpus luteum, the anatomy of the structure has been evalutated and the clinical decision has been made whether it is a compact CL or a CL with a cavity.

After ending the TRUS examination, cows have been released and allowed to go back to their production group into the free stall at the farm.

All clinical findings have been dictated to an assistant who made notes and after a second check what has been described, the findings have been saved. After each weeks' examinations, data have been typed into a table (Microsoft Excel©).
